# Supplementary material for: Soft Multi-Directional Force Sensor for Underwater Robotic Application
Source: Sensors (Basel). 2022 May 19;22(10):3850. doi: 10.3390/s22103850 (PMC9146921; doi:10.3390/s22103850)
Supplement: Supplementary file 1 [file sensors-22-03850-s001.zip › Supplementary_Information.pdf]

Soft Multi-directional Force Sensor for Underwater Robotic Application

Rafsan Al Shafatul Islam Subad †, Md Mahmud Hasan Saikot ‡, and Kihan Park † *

† Department of Mechanical Engineering, University of Massachusetts Dartmouth, 285 Old Westport Road, Dartmouth, MA 02747, USA
‡ Department of Mechanical Engineering, Bangladesh University of Engineering and Technology, Dhaka 1000, Bangladesh

* Correspondence: kihan.park@umassd.edu

Fabrication Process:

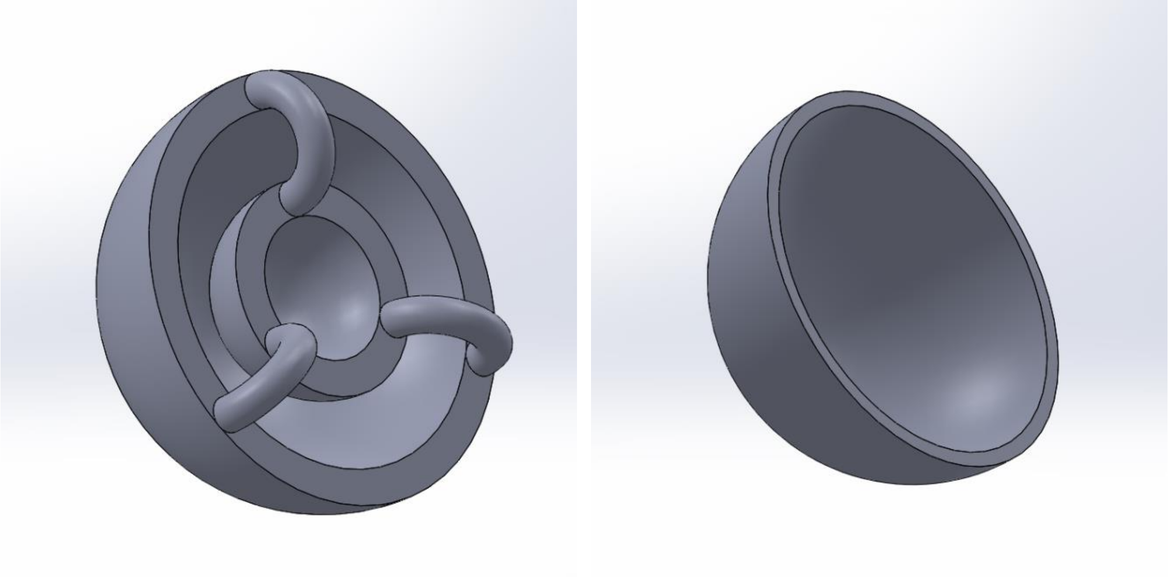

(a) (b)

Figure S1: Image of (a) pattern 1 (b) pattern 2 for the fabrication of the multi-directional force sensor.

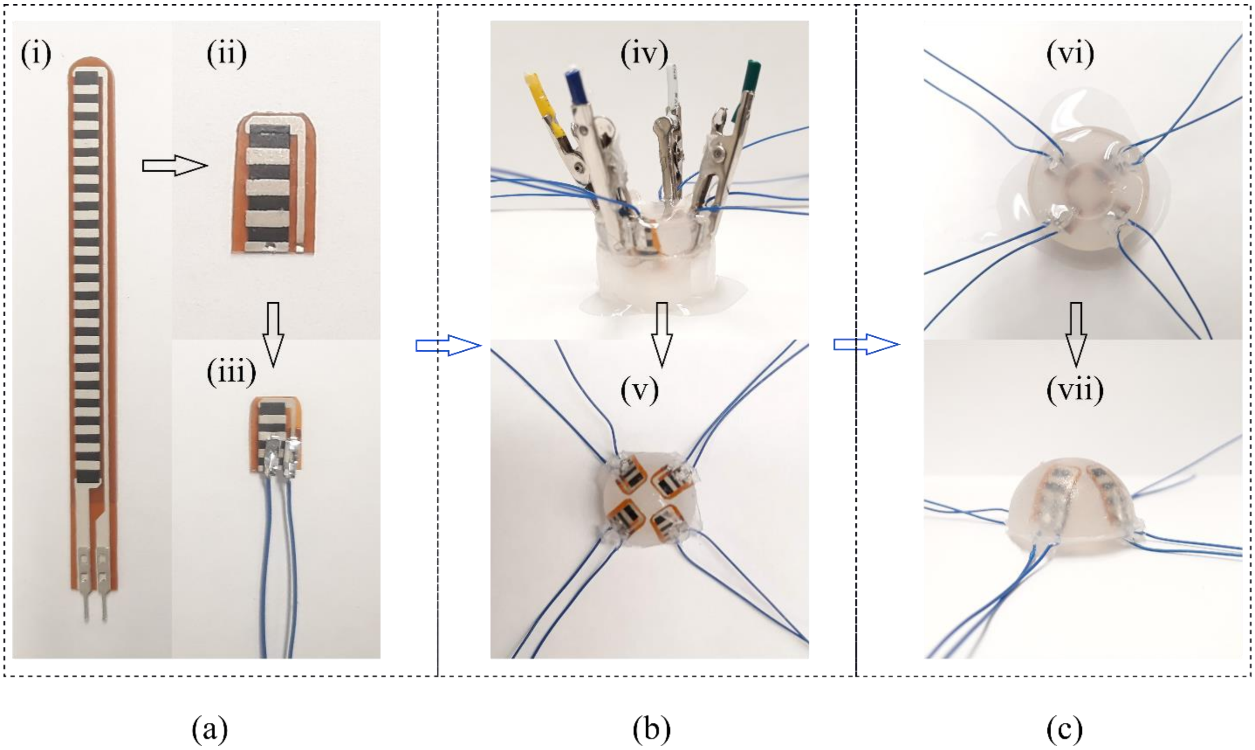

Figure S2: Actual physical images of the step-by-step fabrication process of the sensor (a) preparation of the flex sensor, (b) placing the flex sensors inside the mold with clip and building up initial structure of the sensor (c) formation of the outer layer of the sensor.

Material properties:

Table S1: Properties of the Dragon Skin FX-Pro (silicone material) at 23°C

**Properties**	**Value**
Mixed Viscosity (Pa.s)	18
Specific Gravity (kg/m^3)	1062
Specific Volume (m^3/kg)	0.0009
Shore A Hardness	2
Tensile Strength (kPa)	1985.69
100% Modulus	260.62
Elongation @ Break	763%
Die B Tear Strength(kg-cm)	70.28
Useful Temperature Range (°C)	-53 to +232

Dielectric Strength (volts/mil)	>350
Pot Life (minutes)	12 (at 23°C)
Cure Time (minutes)	40 (at 23°C)

Force Calibration:

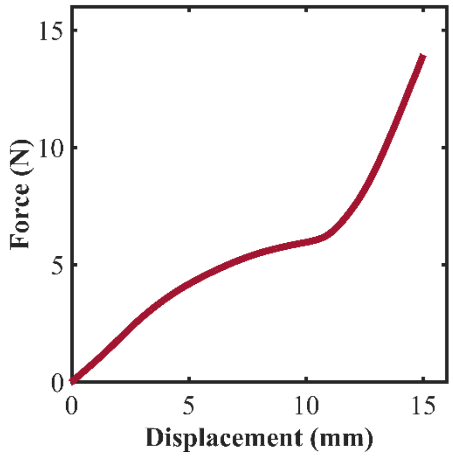

Figure S3: Force-displacement graph for applied normal force on the sensor structure

Repeatability Test:

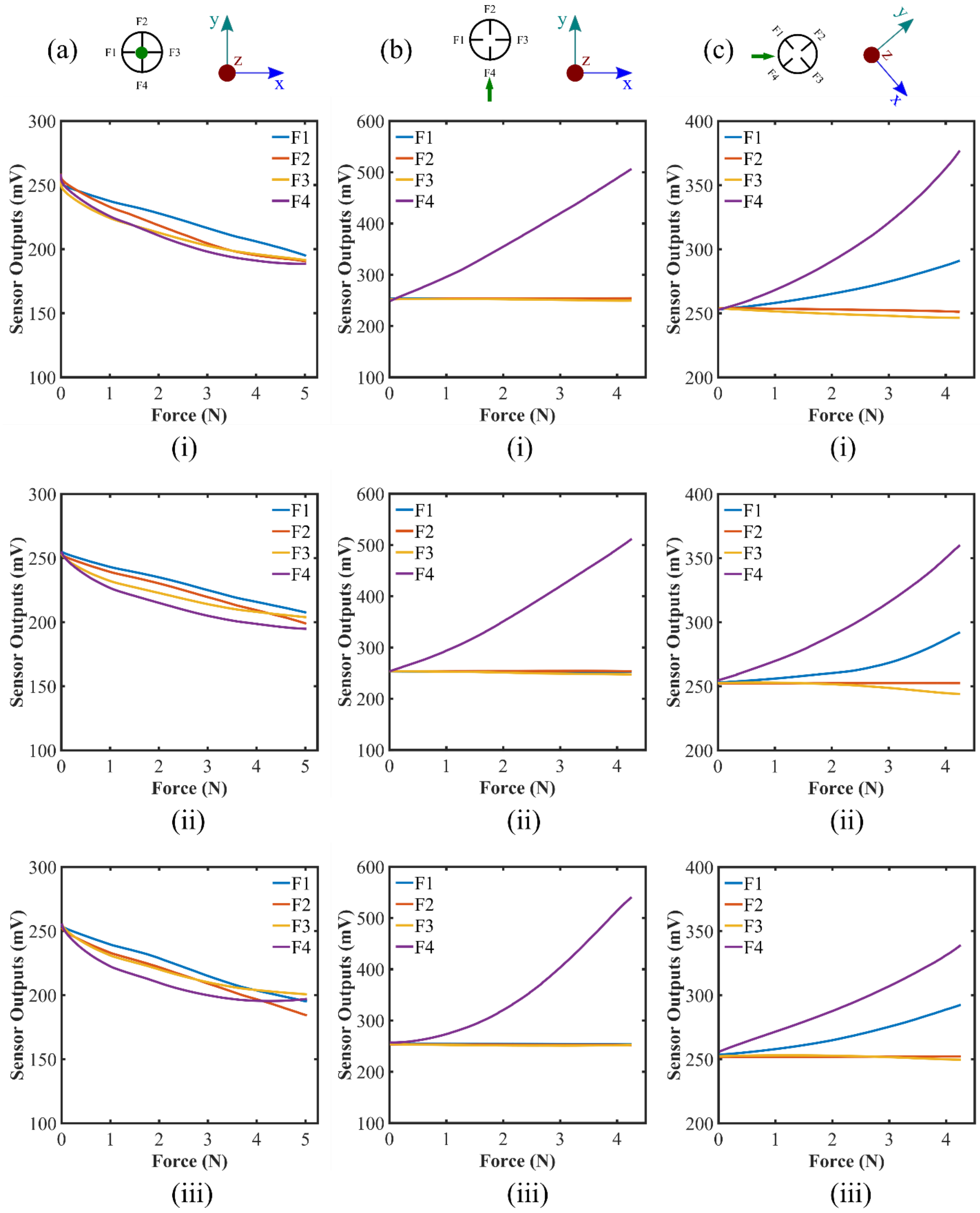

Figure S4: Repeatability test for force calibration for the forces along (a) z-axis, (b) y-axis, and (c) 45° angle from the x-axis; green dot and arrow represent the force direction accordingly; top view of the sensor system is also presented; (i), (ii), and (iii) exhibit trial numbers

Cyclic Loading and Hysteresis Test

Although the force-displacement curves deviate the path a little during a loading-unloading cycle, there is no offset for force or displacement values after the completion of a cycle.

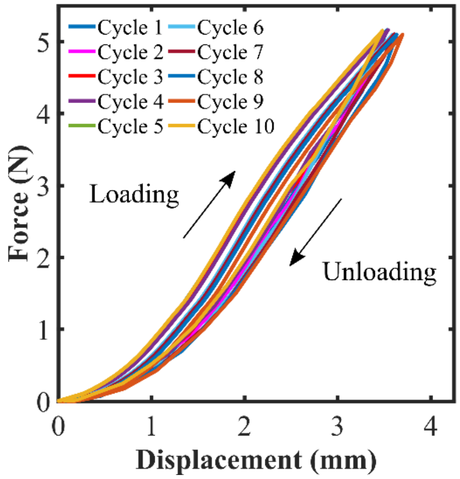

Figure S5. *Force-displacement curves for ten cycles of loading-unloading on the sensor*

Applied Force Location Estimation:

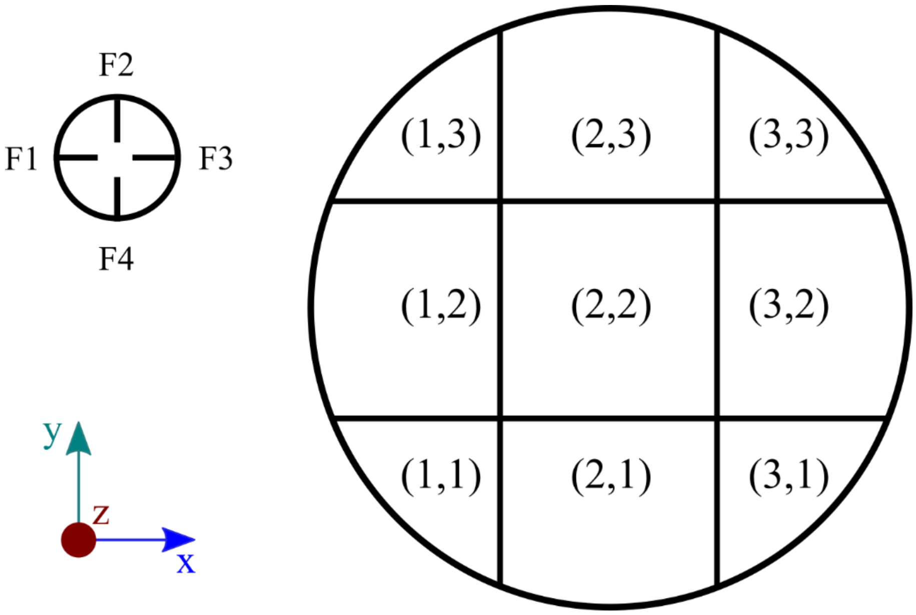

Figure S6: Projection of the hemispherical sensor structure on the xy-plane; a circle divided into a 3×3 grid and the specifications of the grid number; the image in the upper left corner shows the flex sensors' positions, and the lower-left depicts the coordinate system.

Initially, all the values for four flex sensors are assumed to be zero (suppose, a=b=c=d=0 for F1, F2, F3, and F4 accordingly) which means no force. And we set two threshold values, for example, Threshold 1, and Threshold 2. If any of the sensor values pass the defined threshold values, the respective row matrix value of that sensor converts into 1, such as, if Threshold 1<F1>Threshold 2, 'a' becomes 1, and the same criterion goes for b, c, and d values. Possible scenarios of the row matrix and the designated grid respective to the specific row matrix are given below:

Table S2: Row matrix and the assigned grid respective to the specific row matrix for generating applied force locations

Row Matrix	Designated Grid to be Activated
[0 0 0 0]	No grid
[1 0 0 0]	(1,2)
[0 1 0 0]	(2,3)
[0 0 1 0]	(3,2)
[0 0 0 1]	(2,1)
[1 1 0 0]	(1,3)
[0 1 1 0]	(3,3)
[0 0 1 1]	(3,1)
[1 1 1 1]	(2,2)

Underwater Test

The fabricated hemispherical sensor is not enclosed so that the surrounding water would exert the same amount of pressure on both sides of the hemispherical shell. With the water pressure being the same on both sides, the sensor's hemispherical structure would not bend unless any additional force is exerted on the sensor. Thus, the sensor would observe very little or no influence on its outputs by the surrounding water.

However, the sensor size may get squeezed a little or the structure be less flexible under immense water pressure. We haven't been able to test those conditions yet. We have achieved the IP67 standard for waterproofing so far which guarantees waterproofness only for temporary submersion. We'll be able to test those types of conditions once we achieve the IP68 rating for waterproofness (for which we are actively working) in our future scope of the study.
